# Comparison of post-COVID-19 vaccination hypermetabolic lymphadenopathy on ^18^F-fluorodeoxyglucose PET/CT between virus-vector vaccine and mRNA vaccine

**DOI:** 10.1186/s40001-023-01456-1

**Published:** 2023-11-15

**Authors:** Meng-Ting Chiang, Jann-Tay Wang, Wan-Yu Lin, Ruoh-Fang Yen, Jei-Yie Huang, Ching-Chu Lu

**Affiliations:** 1 Department of Nuclear Medicine, National Taiwan University Hospital, No. 7, Zhongshan S. Rd., Zhongzheng Dist., Taipei City, Taiwan; 2 Department of Internal Medicine, National Taiwan University Hospital, Taipei, Taiwan; 3 Institute of Health Data Analytics and Statistics, College of Public Health, National Taiwan University, Taipei, Taiwan

**Keywords:** COVID-19, Virus-vector vaccine, mRNA vaccine, FDG PET/CT, Hypermetabolic lymphadenopathy

## Abstract

**Purpose:**

We compared hypermetabolic lymphadenopathy (HLN) on ^18^F-fluorodeoxyglucose (FDG) positron emission tomography (PET)/computed tomography (CT) after virus-vector and mRNA vaccines for coronavirus disease 2019 (COVID-19).

**Methods:**

This retrospective study included 573 participants who underwent FDG PET/CT after receiving a virus-vector vaccine (ChAdOx1, AstraZeneca [AZ] group) or an mRNA vaccine (mRNA-1273, Moderna [M] group) from July 2021 to October 2021. The incidence and avidity of HLN were evaluated and correlated with clinical features and vaccine type. The final analysis was conducted with 263 participants in the AZ group and 310 participants in the M group.

**Results:**

The HLN incidence was significantly lower in the AZ group than in the M group (38/263 [14%] vs. 74/310 [24%], *p* = 0.006). The FDG avidity of HLN was comparable between the two groups. The HLN incidence in both groups was significantly higher within 4 weeks after the vaccination compared with more than 4 weeks. The HLN incidence within 4 weeks of the vaccination was significantly higher in the M group than in the AZ group (*p* = 0.008), whereas a difference in HLN incidence between the two groups was not observed after the same duration (*p* = 0.11).

**Conclusions:**

The mRNA mRNA-1273 COVID-19 vaccine was found to be associated with higher glucose hypermetabolism in regional lymph nodes within the first 4 weeks compared with the virus-vector vaccine, as indicated by the presence of HLN on FDG PET/CT. The degree of glucose hypermetabolism was comparable between the two vaccines.

## Background

Coronavirus disease 2019 (COVID-19) first occurred in late 2019 and rapidly disseminated across the globe as a pandemic, severely affecting healthcare and economic systems. Effective vaccines against COVID-19 have been developed and globally deployed since early 2021. In Taiwan, the first available COVID-19 vaccine was ChAdOx1, a virus-vector vaccine from Oxford-AstraZeneca (AZ) that was introduced on March 22, 2021. Subsequently, mRNA-1273, an mRNA vaccine from Moderna (M) was introduced on June 12, 2021.

Various studies have reported increased ^18^F-fluorodeoxyglucose (FDG) uptake in axillary lymph nodes and in the administration site on positron emission tomography (PET)/computed tomography (CT) after COVID-19 vaccination [1–16]. Similar findings were noted after influenza and papillomavirus vaccines as well [17–20]. Swelling and pain at the administration site and ipsilateral axillary lymphadenopathy have been reported as side effects of COVID-19 vaccines [21–23]. FDG uptake in the deltoid muscle at the administration side (DL) and hypermetabolic axillary lymphadenopathy (HLN) are expected since local inflammation at the injection site and immune response elicited by vaccination lead to possibly increased glucose metabolism [17–20].

FDG PET/CT is usually performed in oncological patients; thus, differentiating metastatic lymphadenopathy from the benign process may be challenging during the interpretation of HLN. A scientific expert panel suggested that imaging should be postponed for at least 6 weeks after COVID-19 vaccination [24]. Nevertheless, most of the published studies focus on PET findings after mRNA COVID-19 vaccine; these studies originate from countries outside Asia, except for one study in Korea, which included ChAdOx1, and one study in Japan which included Pfizer-BioNTech vaccine BNT162b2 [6, 16].

To date, only one study compared FDG PET uptake patterns among different mRNA vaccines [5]. Therefore, we aimed to evaluate the differences in PET patterns between the virus-vector and mRNA COVID-19 vaccines.

## Methods

### Participants

We conducted a retrospective review of 1578 patients aged ≥ 18 years who underwent FDG PET/CT in National Taiwan University Hospital from July 2021 to October 2021. Among these, 626 participants who received a COVID-19 vaccine before FDG PET/CT were selected for analysis. Data on participants’ age, sex, indication for FDG PET/CT, vaccine type, injection site, date of vaccination, and administration of the first or second dose were reviewed. Participants with insufficient clinical data and those with a known malignancy involving axillary lymph nodes were excluded. The study was approved by an institutional review board at National Taiwan University Hospital (202110071RINB). The requirement to obtain written informed consent from the participants was waived.

### FDG PET/CT acquisition and analysis

FDG PET/CT was performed following the institutional protocol. All participants fasted for at least 4 h and were confirmed to have glucose levels of less than 150 mg before FDG administration. FDG at a dose of 5.29 MBq/kg was intravenously administrated on the opposite side of the vaccination injection site. Oral or intravenous contrast was not administrated. PET/CT was performed 60 min after the FDG administration (GE Discovery PET/CT 710, Milwaukee WI, USA, or Siemens Biograph mCT 20, Erlangen, Germany). A low-dose CT scan from the vertex of the skull to mid-thigh (or feet if indicated) was performed for anatomical localization and attenuation correction of the PET emission data. The PET images were reconstructed by the order subset expectation maximization method using 18 subsets and 3 iterations.

All images were analyzed using commercially available software (syngo.via, Siemens), which allowed the review of PET, CT, and fused imaging data in axial, coronal, and sagittal slices. The interpretation of the images was independently performed by two board-approved nuclear medicine physicians (C.C.L with 16-year PET/CT reading experience and R.F.Y with 32-year PET/CT reading experience) and one nuclear medicine resident (M.T.C) with 2-year PET/CT reading experience. All disagreements were resolved by consensus.

FDG avidity was expressed as maximum standard uptake value (SUVmax) of the axillary lymph nodes and the deltoid muscle on the ipsilateral side of the vaccination by drawing a region of interest at the site of draining axillary lymph nodes and deltoid muscle. HLN and DL were defined as a ratio ≥ 1.5 between the SUVmax of the ipsilateral reference site and the SUVmax of the contralateral reference site [8, 14, 18]. Additionally, systemic response was evaluated using FDG activity in the spleen and bone marrow. A spherical volume-of-interest was set at the center of spleen, center of the right liver lobe (as reference, excluding large vessels and metastatic lesions if present), first lumbar vertebra and right posterior iliac crest [25–27]. Bone marrow/liver SUVmax ratio (BLR) and spleen/liver SUVmax ratio (SLR) were also calculated.

### Statistical analyses

Continuous variables were expressed as means ± standard deviation, and the categorical variables were expressed as frequencies and percentages. Two-sample proportion test was used to compare proportions, and independent *t*-test was used to compare continuous variables. Statistical analysis was performed by *R* software and MedCalc (Ostend, Belgium), and a *p*-value of < 0.05 was deemed to indicate statistical significance.

## Results

During the study period, 626 of the 1578 participants who underwent FDG PET/CT received COVID-19 vaccination. After excluding 16 participants with insufficient clinical data and 37 participants with known malignancy involving the axillary lymph nodes, the remaining 573 participants were included in the final analysis. The cohort included 279 women and 294 men, with a mean age of 64 years. Further, 263 and 310 participants received ChAdOx1 (AZ group) and mRNA-1273 (M group) vaccines, respectively. Table 1 summarizes the demographic and clinical data related to vaccination. Overall, 83% of the participants received the first vaccine (AZ group, 78%; M group, 87%). The most common indication for PET was staging of malignancy (567/573, 99%), with a minority of the participants undergoing imaging for non-oncological conditions (6/573, 1%). Table 2 presents the comparison of the indications for FDG PET/CT between the two vaccine groups.

**Table 1:** Participant characteristics

	Total (*n* = 573)	AZ group (*n* = 263)	M group (*n* = 310)	*p*-value^a^
Age (years)	64 ± 12	62 ± 15	67 ± 9	< 0.0001
Sex, female	279 (49%)	137 (52%)	142 (46%)	0.13
Vaccination dose				0.002
First dose	478 (83%)		271 (87%)	
Second dose	98 (17%)	204 (78%)	39 (13%)	
Injected site				0.78
Left arm	492 (86%)	227 (86%)	265 (85%)	
Right arm	81 (14%)	36 (14%)	45 (15%)	
Time interval between PET and vaccination (days)	51 ± 29	50 ± 30	53 ± 29	0.41

All data were expressed in mean ± SD

AZ, Oxford-AstraZeneca; M, Moderna; PET, positron emission tomography

^a^ Comparison between the AZ and M groups

**Table 2:** Indications of positron emission tomography

	AZ group (*n* = 263)	M group (*n* = 310)	*p*-value
Lung cancer	120 (46%)	137 (44%)	0.74
Hematologic malignancy	54 (21%)	61 (20%)	0.81
Colorectal malignancy	22 (8%)	24 (8%)	0.76
Upper GI malignancy	15 (6%)	24 (8%)	0.34
Head and neck cancer	14 (5%)	17 (6%)	0.92
Hepatobiliary-pancreatic cancer	8 (3%)	9 (3%)	0.94
Breast cancer	7 (3%)	6 (2%)	0.52
Other cancers	15 (6%)	24 (8%)	0.34
Cancer survey	6 (2%)	4 (1%)	0.37
Non-oncologic condition	2 (1%)	4 (1%)	0.56

AZ, Oxford-AstraZeneca; M, Moderna; GI, gastrointestinal

**Fig. 1:**
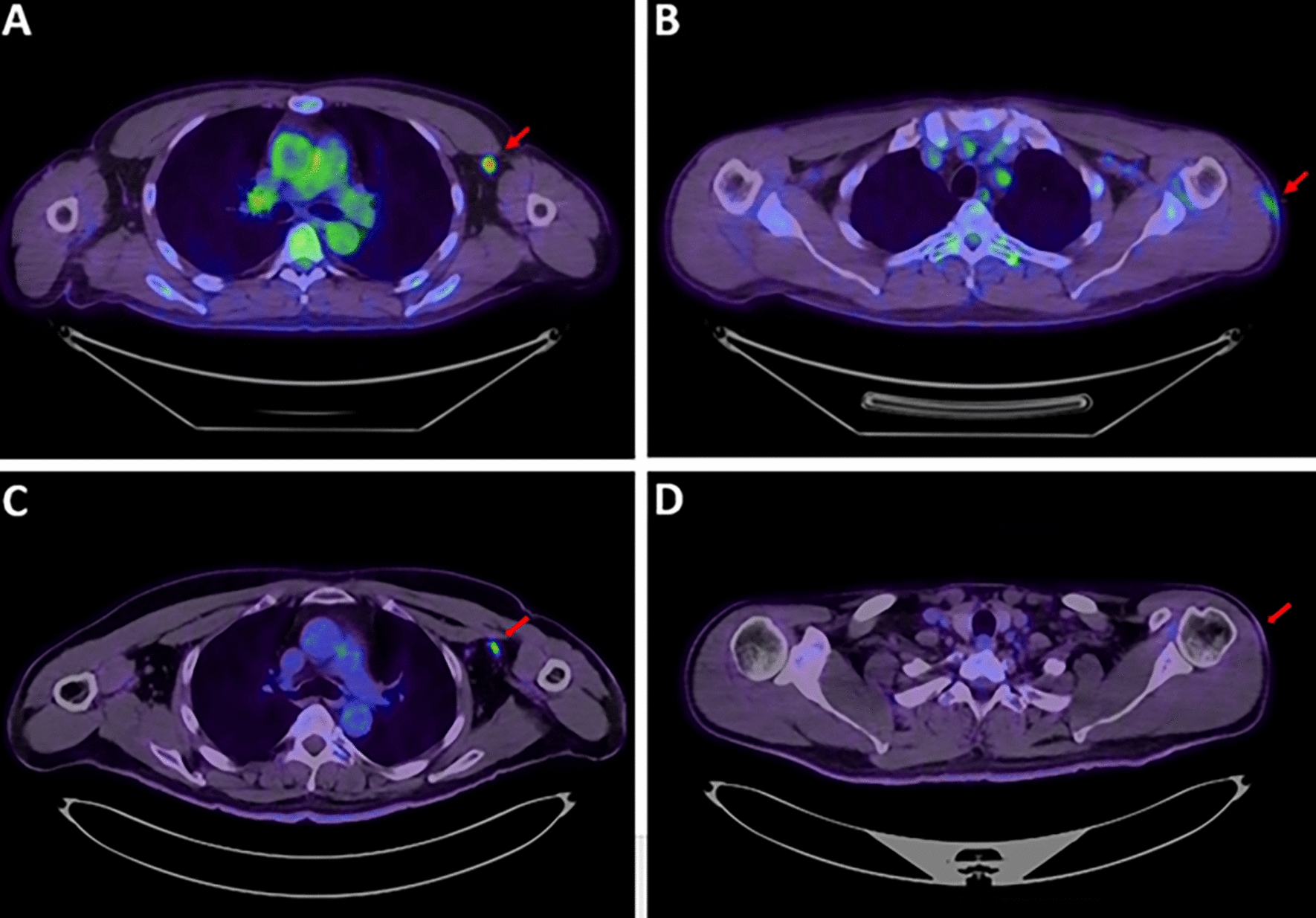
FDG PET/CT after COVID-19 vaccination. **A** and **B** A 42-year-old men underwent FDG-PET/CT for colon cancer staging. He received the first dose of the Oxford-AstraZeneca vaccine in the left arm 6 days before the scan. The SUVmax of HLN was 4.27, and the SUVmax of DL was 1.91. **C** and **D** A 54-year-old men underwent FDG-PET/CT for evaluation of recurrent colon cancer. He had his first dose of Moderna vaccine in the left arm 42 days before the scan. The SUVmax of HLN was 4.48, and the DL showed no hypermetabolism with SUVmax equal to 0.77. FDG PET/CT, ^18^F-fluorodeoxyglucose positron emission tomography/computed tomography; COVID-19, coronavirus disease 2019; HLN, hypermetabolic lymphadenopathy; DL, deltoid muscle uptake; SUVmax, maximal standard uptake value

Table 3 lists the PET patterns in both vaccination groups. The overall HLN incidence was 20% (112/573). HLN was present in 38 of the 263 participants (14%) in the AZ group and seban74 of the 310 participants (24%) in the M group; the HLN incidence was significantly lower in the AZ group than in the M group (*p* = 0.006). The mean SUVmax of HLN was comparable between the two groups (AZ group: 3.0 ± 1.3, M group: 3.0 ± 1.3; *p* = 0.92; Fig. 1). Subgroup analysis according to the vaccination dose revealed that the HLN incidence in participants with the first dose was lower in the AZ group (32/204, 16%) than in the M group (60/271, 22%; *p* = 0.10), although the difference was not statistically significant. The HLN incidence in participants with the second dose was significantly lower in the AZ group (6/59, 10%) than in the M group (14/39, 36%; *p* = 0.005).

**Table 3:** Positron emission tomography patterns in participants receiving different vaccines

	AZ group (*n* = 263)	M group (*n* = 310)	*p*-value
HLN	38 (14%)	74 (24%)	0.006
≤ 4 weeks	21/92 (23%)	39/92 (42%)	0.008
> 4 weeks	17/171 (10%)	35/218 (16%)	0.11
*p*-value^a^	0.008	< 0.0001	
SUVmax of HLN	3.0 ± 1.3	3.0 ± 1.3	0.92
Vaccination dose			
First dose	32/204 (16%)	60/271 (22%)	0.10
Second dose	6/59 (10%)	14/39 (36%)	0.005
*p*-value	0.39	0.09	
DL	27 (10%)	25 (8%)	0.44
≤ 4 weeks	23/92 (25%)	21/92 (23%)	0.86
> 4 weeks	4/171 (2%)	4/218 (2%)	0.73
*p*-value^a^	< 0.0001	< 0.0001	
SUVmax of DL	2.2 ± 0.9	1.9 ± 0.6	0.08

^a^ Comparison between the first 4 weeks and beyond 4 weeks after vaccination

AZ, Oxford-AstraZeneca; M, Moderna; HLN, hypermetabolic lymphadenopathy; DL, deltoid muscle uptake

The overall incidence of DL was 9% (52/573). There was no significant difference in DL incidence between the AZ and M groups (AZ group, 27/263, 10%; M group, 25/310, 8%; *p* = 0.44). The SUVmax of DL was higher in the AZ group than in the M group, albeit with no statistical significance (AZ group, 2.2 ± 0.9; M group, 1.9 ± 0.6; *p* = 0.08).

The HLN incidence was significantly higher than the DL incidence in the M group (HLN, 74/310, 24%; DL, 25/310, 8%; *p* < 0.0001), whereas no difference between the HLN and DL incidence in the AZ group (HLN, 38/263, 14%; DL, 27/263, 10%; *p* = 0.19). The FDG uptake was significantly higher in the axillary lymph nodes than in the deltoid muscle in both groups (AZ group, 3.0 ± 1.3 vs. 2.2 ± 0.9, *p* = 0.01; M group, 3.0 ± 1.3 vs. 1.9 ± 0.6, *p* = 0.0001). The SUVmax of the DL was higher in the AZ group than in the M group (AZ group, 2.2 ± 0.9; M group, 1.9 ± 0.6, *p* = 0.08), albeit without statistical significance.

The average time interval between the FDG PET/CT and vaccination was 50 ± 30 days in the AZ group and 53 ± 29 days in the M group (*p* = 0.41). The incidence of HLN and DL was inversely correlated with the time interval. In both groups, the HLN incidence was significantly higher within the 4 weeks after vaccination than after the 4 weeks of vaccination (AZ group, 23% vs. 10%, *p* = 0.008; M group, 42% vs. 16%, *p* < 0.0001). Similarly, the DL incidence was higher within the first 4 weeks after vaccination than after the 4 weeks of vaccination in both groups (AZ group, 25% vs. 2%, *p* < 0.0001; M group, 23% vs. 2%, *p* < 0.0001).

There were no differences in terms of BLR and SLR of the AZ and M groups (BLR, AZ group, 0.65 ± 0.20; M group, 0.63 ± 0.19, *p* = 0.90; SLR, AZ: 0.83 ± 0.46; M, 0.81 ± 0.12, *p* = 0.52). Subgroup analysis using the 4-week time interval between the FDG PET/CT and the last vaccination as a cutoff revealed no significant differences in BLR and ALR between the groups.

## Discussion

In the present study, the HLN incidence was significantly higher in participants who received the mRNA vaccine than those who received the virus-vector vaccine, although the HLN avidity was comparable between the two groups. These findings implicate a higher rate of glucose hypermetabolism in regional lymph nodes associated with the mRNA vaccine compared with the virus-vector vaccine despite similar metabolic responses. Increased glucose metabolism of lymph nodes might be associated with immune response triggered by vaccination. B cells exhibit a metabolic shift from fatty acid oxidation to glycolysis during activation through various pathways such as the direct binding of HIF-1α and c-Myc to the promoters of genes encoding glycolytic enzymes and glucose transporters [28, 29]. B cell proliferation and the production of high-affinity antibodies demand high energy, and several studies have reported that B cells in germinal centers exhibited increased glucose consumption compared with naïve B cells [30–32]. Our results further supported the previously implicated molecular pathways by PET findings. Most studies on COVID-19 vaccinations reported that the HLN incidence related to mRNA vaccines ranged from 13 to 69% (Table 4). The only Korean study investigating HLN after the AZ COVID-19 vaccine reported an incidence of 90% among participants who were considered as otherwise healthy [6]. The mean SUVmax of HLN was significantly lower in the present study compared with the other Korean study (2.2 ± 0.9 vs. 3.9 ± 1.7, *p* < 0.0001), based on the data provided in that article. One potential reason for this difference is that 99% of the current study cohort were oncological patients. Cohen et al. demonstrated that the HLN incidence was lower after the two doses of an mRNA vaccine in hematological patients with suppressed immune status confirmed by serology [4]. Eifer et al. reported that the HLN incidence is inversely associated with older age, immunosuppressive treatment, and the presence of hematological disease [8]. Seban et al. also reported that the absence of lymphopenia, age ≤ 50 years, and longer intervals between vaccination and FDG PET/CT were independent predictors of HLN in patients with breast cancer [14]. Patients with cancer may have altered immune systems, leading to an attenuated response to vaccination, which might explain the lower HLN incidence observed in the current cohort compared with the Korean study. However, larger cohorts of healthy subjects and a more detailed understanding of the underlying mechanisms are needed for further investigations. Our study reveals that HLN caused by vector vaccines has a lower incidence in the first 4 weeks than with mRNA vaccines. This may influence future vaccine choices for specific patient groups such as in oncological conditions with higher chance of axillary lymph node involvement. The significant drop in HLN incidence after 4 weeks also informs optimal timing for imaging assessments in cancer patients to distinguish disease progression from HLN. Currently, the mechanism underlying the difference of HLN incidence remains largely obscured. However, it is noteworthy that mRNA vaccines directly encapsulate their payload within polyethylene glycol, whereas vector vaccines rely on inactivated vector viruses for their function. It is conceivable that this disparity in the vaccination process might lead to a comparatively diminished locally mediated immune response in the case of vector vaccines. Nevertheless, it is imperative to acknowledge that further extensive research is imperative to substantiate these conjectures.

**Table 4:** Positron emission tomography patterns in the reported studies pertaining to COVID-19 vaccination

Country	Case	Vaccine	HLN(%)	HLN1(%)^a^	HLN2(%)^a^	HLN SUV^b^	HLN SUV1^ab^	HLN SUV2^ab^	DL (%)	DL1 (%)^a^	DL2 (%)^a^	DL SUV^b^	DL SUV1^ab^	DL SUV2^ab^
Israel [1]	650	BNT	26	15	43		3.7	4.5						
Israel [2]	951	BNT	46	36	54									
Israel [3]	205	BNT	29				2.9							
Israel [4]	137	BNT	31											
Switzerland [5]	140	BNT/M	54			5.1	4.1	5.4						
Korea [6]	31	AZ	90			3.9			73			2.8		
USA [7]	68	BNT/M	13	5	26									
Israel [8]	377	mRNA	45			2.7			26			2.0		
USA [9]	231	mRNA	47									1.8		
Israel/UK [10]	274	NA	66	55	69	0.6–17.8^c^			12					
Italy [11]	437	BNT/M/AZ	27	34	66	4.1								
Israel [12]	179	BNT	48			2.4								
UK [13]	204	BNT/AZ	36			1.9								
France [14]	260	mRNA	35			3.7								
Canada [15]	202	mRNA	62			2.5								
Japan [16]	237	BNT	43	41	51		2.9	1.8	22	19	34		1.8	2.2

HLN, hypermetabolic lymphadenopathy; DL, deltoid muscle uptake; BNT, Pfizer-BioNTech; M, Moderna; AZ, Oxford-AstraZeneca

^a^ HLN1, HLN incidence after the first dose; HLN2, HLN incidence after the second dose; DL1, DL incidence after the first dose; DL2, DL incidence after the second dose

^b^ SUV represented for maximal standard uptake value

^c^ Range

Regional lymphadenopathy was reported in 0.3% of the recipients after the BNT162B2 vaccine, whose package leaflet classifies enlarged lymph nodes as an uncommon side effect [33]. The incidence of lymphadenopathy as an adverse effect was higher in participants who received the mRNA-1373 vaccine compared with the placebo group. Reports of lymphadenopathy were imbalanced, with 1.1% of subjects in the vaccine group and 0.6% of subjects in the placebo group. The government of the United Kingdom reported a lymphadenopathy incidence of 0.01% in the general British population [34]. A subjective palpable, painful, or exteriorly enlarged lymph node is deemed as lymphadenopathy induced by vaccination, whereas FDG PET/CT is an indirect method to measure glucose metabolism in lymph nodes, which partially explains the significant difference between the reported incidence of lymphadenopathy as a side effect and the incidence of HLN.

The antiviral effect of vaccination includes a cellular response achieved by cytotoxic T cells, followed by humoral response mediated through antibody-secreting plasma cells and memory B cells in the germinal centers of lymph nodes [35]. Ukey et al. evaluated the immunogenicity of virus-vector and mRNA vaccines and reported that the mRNA vaccines exhibited higher humoral response, based on anti-SARS-CoV-2 receptor-binding-domain IgG antibodies and neutralizing titers calculated as NT50 (reciprocal dilution of plasma yielding 50% neutralization of live SARS-CoV-2 virus), compared with the virus-vector vaccines [36]. This difference might partially explain the higher HLN incidence in the M group compared with the AZ group observed in the present study. Lederer et al. reported that the humoral response was stronger with the mRNA vaccine than with the recombinant protein vaccine [37]. The HLN incidence is predicted to be higher with mRNA vaccines than with the recombinant protein vaccines. Evaluating vaccine immunogenicity becomes more important after mass vaccination. In their report of a patient with systemic inflammatory response syndrome after COVID-19 vaccination, Steinberg et al. illustrated that FDG PET/CT features may be essential for assessing immune response besides laboratory findings [38]. However, we observed no difference in FDG avidity in the liver and bone marrow of the mRNA and virus-vector vaccines, suggesting that HLN is a regional reaction instead of a systemic response.

Burger et al. reported that 17 of 58 (29%) participants who received the H1N1 influenza vaccine had HLN with a mean SUVmax of 2.4 [19]. We could not discern a significant difference in HLN incidence between the H1N1 influenza vaccine and either the virus-vector or the mRNA COVID-19 vaccine (Table 5). The avidity of HLN appears to be comparable in participants who receive a COVID-19 vaccine compared to those who receive the H1N1 influenza vaccine. Therefore, it appears that the increased glucose metabolism in lymph nodes triggered by the COVID-19 and vaccines H1N1 influenza vaccines may be comparable.

**Table 5:** Comparison of positron emission tomography patterns between the Oxford-AstraZeneca and Moderna COVID-19 vaccines and the H1N1 influenza vaccine ([19])

Vaccine type	HLN incidence %	HLN SUVmax	DL incidence	DL SUVmax
ChAdOx1	14	3.0 ± 1.3^a^	10%	2.2 ± 0.9
mRNA-1237	24	3.0 ± 1.3^b^	8%	1.9 ± 0.6
H1N1	29	2.4 ± 1.1	NA^c^	2.9 ± 1.2

HLN, hypermetabolic lymphadenopathy; DL, deltoid muscle uptake

^a^ ChAdOx1 vs. H1N1, *p* = 0.11

^b^ mRNA-1237 vs. H1N1, *p* = 0.09

^c^ NA: not available

The present study has several limitations. First, this was a retrospective study including a highly selective oncological population. Nevertheless, the PET patterns that were different from those reported in healthy workers in a Korean study reflect a potentially variable glucose metabolism in regional lymph nodes among specific populations. Second, the current study findings do not provide direct evidence of a relationship between glucose metabolism in regional lymph nodes and immune response measured by serological tests. Third, most of the study participants received the first dose of the COVID-19 vaccine. Further studies are warranted following the global implementation of the second and booster doses. Fourth, the lack of histological assessment for HLN precluded the assessment of malignancy in lymph nodes. Nevertheless, the history of vaccination together with distinct PET patterns, especially the double sign reported by Orevi et al., is highly suggestive of HLN rather than malignancy [10].

## Conclusions

HLN was more frequent in participants who received an mRNA COVID-19 vaccine compared to those who received a virus-vector COVID-19 vaccine; however, the degree of glucose hypermetabolism was similar in both recipient groups.

## Data Availability

All data generated or analyzed during this study are included in this article, and materials are available from the corresponding author upon reasonable request.

## Abbreviations

HLN: Hypermetabolic lymphadenopathy
FDG: 18F-fluorodeoxyglucose
PET: Positron emission tomography
CT: Computed tomography
COVID-19: Coronavirus disease 2019
AZ: AstraZeneca
M: Moderna
DL: FDG uptake in the deltoid muscle at the administration side
SUVmax: Maximal standard uptake value
BLR: Bone marrow/liver SUVmax ratio
SLR: Spleen/liver SUVmax ratio
